# Agency responses to a system-driven implementation of multiple evidence-based practices in children’s mental health services

**DOI:** 10.1186/s12913-017-2613-5

**Published:** 2017-09-19

**Authors:** Jennifer Regan, Anna S. Lau, Miya Barnett, Nicole Stadnick, Alison Hamilton, Keri Pesanti, Lillian Bando, Lauren Brookman-Frazee

**Affiliations:** 1 Department of Psychology, University of California, Los Angeles, Los Angeles, CA 90095 USA; 2 Department of Counseling, Clinical, and School Psychology, Gevirtz Graduate School of Education, University of California, Santa Barbara, Santa Barbara, CA 93106 USA; 3 Department of Psychiatry, University of California, San Diego, La Jolla, CA 92093 USA; 4 Department of Psychiatry and Biobehavioral Sciences, David Geffen School of Medicine, University of California, Los Angeles, Los Angeles, CA 90095 USA; 5 Mental Health Services Act - Prevention and Early Intervention Administration Division, Program Support Bureau, Los Angeles County Department of Mental Health, Los Angeles, CA 90020 USA; 6 Child and Adolescent Services Research Center, San Diego, CA 92123 USA

**Keywords:** Children’s Mental health, Fiscally-driven implementation, Qualitative methods, Implementation strategies

## Abstract

**Background:**

Large mental health systems are increasingly using fiscal policies to encourage the implementation of multiple evidence-based practices (EBPs). Although many implementation strategies have been identified, little is known about the types and impacts of strategies that are used by organizations within implementation as usual. This study examined organizational-level responses to a fiscally-driven, rapid, and large scale EBP implementation in children’s mental health within the Los Angeles County Department of Mental Health.

**Methods:**

Qualitative methods using the principles of grounded theory were used to characterize the responses of 83 community-based agencies to the implementation effort using documentation from site visits conducted 2 years post reform.

**Results:**

Findings indicated that agencies perceived the rapid system-driven implementation to have both positive and negative organizational impacts. Identified challenges were primarily related to system implementation requirements rather than to characteristics of specific EBPs. Agencies employed a variety of implementation strategies in response to the system-driven implementation, with agency size associated with implementation strategies used. Moderate- and large-sized agencies were more likely than small agencies to have employed systematic strategies at multiple levels (i.e., organization, therapist, client) to support implementation.

**Conclusions:**

These findings are among the first to characterize organizational variability in response to system-driven implementation and suggest ways that implementation interventions might be tailored by organizational characteristics.

## Background

### System-driven implementation of multiple EBPs

Efforts to implement evidence-based practices (EBPs) in community mental health systems have increased considerably [1, 2], with several public mental health systems instituting fiscal policies to support EBP delivery [3]. In Philadelphia, the city mental health system uses a request for proposal process to provide funding supporting EBP implementation with fiscal incentives for certain EBPs [4]. In Los Angeles County, the Department of Mental Health (LACDMH) enacted a plan to utilize a state revenue stream from a voter-approved ballot initiative, the Mental Health Services Act (MHSA), to promote the use of EBPs through new contracts for Prevention and Early Intervention (PEI) services [5, 6]. The EBP implementation was linked with two other major shifts in service provision: (1) expansion to a new target population (i.e., early in the course of mental illness) and (2) requirement for the collection of clinical outcome measures linked to individual EBPs.

The MHSA was passed by voters in 2004 and each county was responsible for developing a PEI plan for state approval. Following a lengthy stakeholder-engaged planning process, the State approved LACDMH’s PEI plan in August, 2009, which included a systematic roll-out of 52 approved evidence-based, promising and community-defined, evidence-informed practices [7]. However, approximately 5 months later, the state budget crisis resulted in an LACDMH budget shortfall estimated between $32–42 million, which could have resulted in a devastating cut in service delivery impacting all provider agencies in Fiscal year ‘10–11. Agencies contracted with LACDMH were expected to lose 50% to 100% of their funding allocation under County General Funds, which threatened the viability of their continued operation. In the context of this urgent financial crisis and to prevent the imminent closure of community-based agencies and substantial reduction in the number of clients served, LACDMH transformed and accelerated the PEI implementation to allow agencies to leverage MHSA funds.

**Fig. 1:**
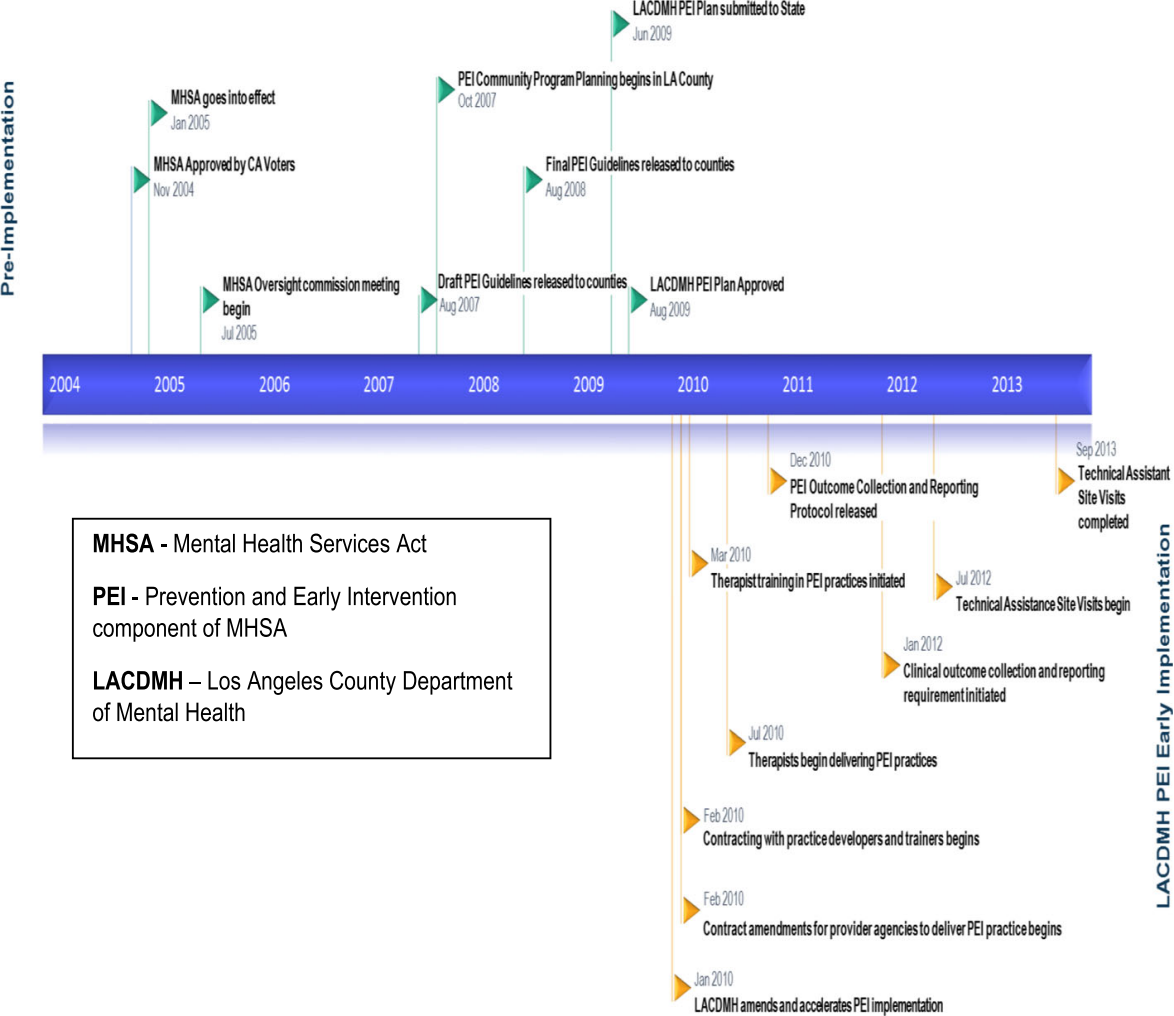
Timeline of major mental health services act-related events in Los Angeles County 2004-2013

In May 2010, LACDMH facilitated the rapid launch of an initial set of six evidence-based/informed practices (hereafter referred to as “practices”) to address a range of prevalent youth mental health problems, including Cognitive Behavioral Intervention for Trauma in Schools [CBITS], Child-Parent Psychotherapy [CPP], Managing and Adapting Practice [MAP], Seeking Safety [SS], Trauma-Focused Cognitive Behavioral Therapy [TF-CBT], and Triple P Positive Parenting Program [Triple P] (See Table 1). These practices were selected by LACDMH due to their wide range of presenting problem areas and the ability of practice developers to rapidly train a large number of staff. Consistent with the MHSA county requirements, LACDMH provided implementation support (i.e. training, consultation, implementation guidelines, and technical assistance). A timeline of major events across phases of PEI implementation in LACDMH is presented in Fig. 1 to highlight the rapid and accelerated nature of the system-driven implementation. This paper describes agencies’ experiences at the outset of the PEI implementation and the strategies they employed to support EBP implementation.

**Table 1:** Characteristics of the six PEI practices

Practice	Age range served	Presenting problems	Required training protocol	Train-the-trainer model
Child-Parent Psychotherapy (CPP) [36]	0–6	Trauma, Attachment	Initial training, 6- and 12-month booster training, bi-weekly group consultation for 18 months	No
Cognitive Behavioral Intervention for Trauma in Schools (CBITS) [37]	10–15	Trauma	On-site initial training, weekly consultation for at least one 10-week group cycle	Yes
Managing and Adapting Practice (MAP) [38]	0–21	Depression, Anxiety, Trauma, Conduct	Initial training, 12 hours of consultation over the course of 6 months, successful portfolio submission	Yes
Seeking Safety (SS) [39]	13–20	Trauma, Substance Use	Initial Training	Yes
Trauma-Focused Cognitive Behavioral Therapy (TF-CBT) [40]	3–18	Trauma	Initial online and in-person training, 16 consultation calls, booster training, submit up to 2 audio taped sessions to certified trainer for review, per the audio tape protocol.	No
Triple P Positive Parenting Program [41]	0–18	Conduct	Initial training, obtain accreditation	No

### System-driven implementation strategies

Understanding EBP implementation in routine care requires attention to both the interventions to be deployed and the implementation strategies used to facilitate their deployment. Powell and colleagues [8] used a modified Delphi method to identify and define 73 implementation strategies and applied concept mapping to organize the strategies into distinct categories [9]. Consistent with the EPIS implementation framework [10] distinguishing multiple phases of implementation (i.e., Exploration, Preparation, Implementation, Sustainment) and multiple levels of influences (i.e., outer context and inner contexts), the strategies may be employed at different implementation phases and levels. System-driven implementation efforts are unique in that strategies are likely employed concurrently in both the outer and inner contexts. Regarding the outer context, LACDMH employed a number of system-level implementation strategies identified by these studies [8, 9] primarily in the categories of *utilizing financial strategies, developing stakeholder relationships, using evaluative and iterative strategies, and providing interactive assistance.* The specific strategies included: *fund and contract for the clinical innovation* (i.e., rapidly amending contacts to deliver approved PEI practices in the context of a fiscal crisis), *place innovation on formularies* (i.e., claiming linked to individual practices), *centralize technical assistance* (i.e., furnishing initial training/consultation for specific practices for over 3300 therapists during the first year)*, develop a formal implementation blueprint* (i.e., ongoing, iterative development of PEI Guidelines based on providers’ initial experiences), *develop and implement tools for quality monitoring*/*provide local technical assistance* (e.g., conducting Technical Assistance Site Visits), *promote network weaving* (e.g., Learning Networks) and *use advisory boards and workgroups* (i.e., community partner and LACDMH staff workgroups proposed PEI guidelines)*.*

Some of the strategies were part of the initial, state-approved PEI Implementation Plan and developed through the stakeholder-involved planning process (e.g., the formulary for 52 approved practices). Other strategies were employed during the acute transformation prompted by the fiscal crisis (e.g., selecting six practices which had the capacity to quickly roll out trainings to a large workforce). The remaining strategies were developed in response to challenges and needs that arose during the initial phase of implementation (e.g., development of the *PEI Implementation Handbook* [11]). Use of these multiple system-level strategies contributed to the outer context of EBP implementation across organizations and practices.

### Organizational characteristics and implementation processes and outcomes

Although the outer context and system-level implementations strategies may be held constant across the agencies, individual organizations may vary in response based on their own contexts and characteristics. Research has identified several inner-context organizational factors that influence implementation. Having a supportive and open organizational climate has been linked to lower staff turnover and greater sustainment of practices [12, 13]. Organizational processes, such as shared decision-making and communication both within and between organizations, play an important role in positive implementation outcomes [14, 15].

In contrast, structural characteristics of agencies have not been as well-studied. An early review identified structural characteristics, including organizational size, functional differentiation and specialization (i.e., number of organizational units and specialties) associated with adopting innovations in healthcare settings [16]. However, organizational size may be a proxy for more nuanced factors, such as having more slack resources to devote to new initiatives resulting in a more receptive context. It is unclear how agency size itself may relate to EBP implementation experiences as it could either facilitate or inhibit implementation support. It is possible that large-size agencies have more resources and, thus, more capacity to mount effective implementation strategies. Yet, it is also possible that larger agencies may have greater bureaucracy that constrains innovative responses to new initiatives. Smaller-size agencies, on the other hand, may have fewer resources, but may be more able to nimbly respond to change due to decreased bureaucracy. Understanding the role of agency size may inform ways to match implementation strategies to specific organizational profiles. Use of implementation strategies may also differ in terms of agency bureaucratic structure, which may be indicated by the extent to which organizational administration and operations are centralized versus dispersed across units. It is plausible that the process of EBP implementation may be more complex in agencies where providers are spread out among multiple sites with varying structures and cultures.

### Organizational implementation strategies

Although there is growing research on leader and therapists’ perceptions of EBPs [17, 18] and on organizational outcomes of EBP implementation efforts (e.g., reduced staff turnover [19], improved implementation climate [20]), there is less research on how organizational leaders perceive and respond to system-level implementation strategies. The limited existing research indicates that key implementation strategies to facilitate success include stakeholder outreach and stakeholder-engaged planning, attending to short- and long-term implementation costs, and focusing on the scalability of EBP training [18, 21].

Few studies have examined strategies agency leaders have independently put into place following a large-scale system reform [22]. It is not known which implementation strategies agencies employ in response to system-driven implementation, and the impact of system-level implementation strategies on organizations. Previous research has shown that different stakeholder groups (i.e., system leaders, agency leaders, front-line staff, treatment developers) have unique priorities and goals related to EBP implementation [23, 24] and these differences can be perceived as barriers [18]. In one study [18], implementation barriers and facilitators were shared across stakeholder groups and concentrated around specific inner context, outer context, and intervention factors (e.g., financing, therapist-level characteristics). However, the relative emphasis on each of these levels varied across stakeholder groups (e.g., system leaders were more concerned with the outer context, agency leaders with the inner context and intervention factors), with stakeholder priorities shaped by factors most proximal to them.

### Present study

This qualitative study provides an in-depth examination of organizational-level responses to a rapid, fiscally-driven EBP implementation in children’s mental health within the LACDMH PEI Transformation. Qualitative methods were used to characterize the responses of 83 community-based agencies to the system-wide implementation of multiple practices based on archived materials from site visits conducted 2 years after the PEI Transformation began. Documents from these site visits offer a view into the inner context of organizations undergoing a large-scale operations change. We examined site visit documentation to characterize the perceptions of agency leaders and system leaders concerning the use of specific organization-level implementation strategies and perceptions of the impact of system-level implementation strategies during the initial implementation of PEI practices. Lastly, we examined whether organizational structural characteristics were associated with the types of implementation strategies agencies used in this context.

## Methods

### Sample

As of August 2012, 124 agencies were contracted to provide PEI services and 119 site visits were conducted. Inclusion for the current analyses involved agencies providing PEI services to children or transition-age youth (TAY) in outpatient settings. Documents for 36 agencies were excluded, 19 based on age of population served and 15 based on service setting (i.e., agencies providing residential care only). Documents for 83 agencies were included.

Two structural agency characteristics were extracted from utilization management reports. *Agency size* was indexed by the average number of child clients (*M* = 368, *SD* = 461, range 0 to 2347) and TAY clients (*M* = 104, *SD* = 146, range 0 to 1056) served per agency within the fiscal year of the site visit (2011-12 or 2012-13). Based on these figures, agencies were categorized into those that served fewer child or TAY clients (small, < 100 child clients, *N* = 23; < 25 TAY clients, *N* = 23), those that served a moderate number of child or TAY clients (moderate, between 100 and 500 child clients, *N* = 42, between 25 and 120 TAY clients, *N* = 33), and those that served a significant number of child or TAY clients (large, > 500 child clients, *N* = 17; > 120 TAY clients, *N* = 26). *Agency centralization* was based on whether agencies had a single program site (55%) or more than one program site (45%).

### Data sources and extraction

The LACDMH PEI Implementation Unit conducted site visits with each PEI-contracted agency from August 2012 to September 2013. The purpose of these visits was to assess implementation milestones (e.g., practitioner training/credentialing, outcome tracking) and provide technical assistance for compliance with system-level functions.

1. **Utilization Management Reports.** Prior to the site visits, LACDMH provided each agency with a utilization management report detailing client demographics, PEI allocations and claims for fiscal years 2010-11, 2011-12 and/or 2012-13, and outcome measurement compliance by practice. Agency characteristics, including size and cost per client, were based on data from these reports.
2. **PEI Technical Assistance Site Visit Provider Pre-Site Visit Questionnaire (PSVQ).** An agency leader or program manager completed the mandatory PEI Provider PSVQ prior to their site visit. The PSVQ included specific questions related to: 1) plans for practices, 2) outcome measurement, 3) PEI implementation successes, 4) PEI implementation challenges, 5) unique infrastructure created to facilitate PEI implementation, 6) fidelity monitoring, 7) plans for sustainment, 8) practice-specific challenges/questions, and 9) training/technical assistance needs.
3. **PEI Technical Assistance Site Visit Summary Report (TASV).** Site visits included a three-hour meeting led by LACDMH staff and attended by agency program managers, supervisors, and therapists. Regarding individual informants, a range of two to 15 agency representatives attended their agency’s site visit (559 in total) and a range of two to 11 system leaders (71 total) attended each meeting. One or two of the four consultants was present for each site visit meeting and consolidated their findings into the site visit reports. A third-party company produced summary reports within 3 weeks of each visit. Each TASV included five standard components: 1) site visit participant information, 2) characteristics of the agency and an overview of PEI implementation, 3) strengths and successes, 4) challenges and concerns, 5) next steps and follow-up actions.

### Data analysis

A methodology of “Coding Consensus, Co-occurrence, and Comparison” [25] rooted in principles of grounded theory [26] was used to code qualitative data from the TASV reports and the PSVQs. All documents were analyzed using ATLAS.ti (ATLAS.ti Scientific Software Development, version 7.5.6; http://atlasti.com/). The initial coding team (A.H., M.B., J.R., & N.S.) independently reviewed six TASV reports and developed inductive, descriptive codes to categorize the content of the reports. After discussion about the clarity, comprehensiveness, and distinctiveness of the codes, the team agreed upon 32 codes (e.g., claiming, client severity, outcome measures, adaptations) and corresponding definitions. This revised list of 32 codes was applied to approximately 20% of the reports, which resulted in the addition of six inductive codes (e.g., treatment length, documentation, non-PEI practice). After independent coding of additional site visit reports using the revised code list, the initial coding team evidenced high agreement and reached consensus resulting in a final coding scheme of 38 codes (available upon request). Two coders were added to the team to complete coding of all documents and evidenced high agreement with the initial six coded TASVs. The full coding team individually applied the final coding scheme to the remaining reports. As the content of the PSVQs was very similar to the TASVs, the same coding system and methodology was applied.

Once all documents were coded, individual codes were clustered into what ATLAS terms “code families” that captured similar content to identify emergent themes (e.g., PEI implementation requirements, organizational response, clinical delivery of practices). Themes were determined to reflect agency or system perspectives according to which party was referenced in relation to that theme in the TASV text. All comments in the PSVQs were attributed to agencies. The role of agency characteristics was examined by creating ATLAS “document families,” or clusters of documents based on key characteristics. These families allowed us to compare and contrast code content within and across document families. For this analysis, we examined two structural agency characteristics: agency size and agency centralization. Document families for these two characteristics were unique (i.e., small agencies could have multiple sites, moderate or large agencies could be single-site). Themes regarding agency structural characteristics were considered to differ substantially from one another if the theme occurred considerably more frequently (determined by consensus) in one document family as opposed to the comparison families.

## Results

Themes that emerged from both data sources were grouped into the following three categories: 1) Perceptions of System Implementation Strategies, 2) Perceptions of PEI Practice Implementation, and 3) Types of Agency Implementation Strategies. See Table 2 for illustrative quotes for each theme. Quotes in the text are annotated according to initial category, which is denoted by number; theme, which is denoted by letter; and then subtheme, which is denoted by Roman numeral. For example, 1ai corresponds to category 1: Perceptions of System Implementation Strategies, theme a: Important role of Training/ technical assistance, and subtheme i: Limited availability of training.

**Table 2:** Themes and representative quotes

**1. Perceptions of System Implementation Strategies**	
**a. Important role of Training/technical assistance**	**i. Limited availability of training (Agency Leaders).** *“Training in a practice being available when it is needed. This has been a major issue with some practices. Certainly when you have the resignation of a fully trained staff, it has an impact.”*
	**ii. Training in multiple practices considered positive achievement (Agency Leaders).** *“Most of our current clinical staff are trained in an average of 3 Practices. We have done this over a period of 2 years. This gives us much greater flexibility with assigning cases and increasing access to care for clients.”*
	**iii. Need for ongoing funding for training (Agency Leaders).** *“(1) Financial support of Train-the-Trainer in PCIT. (2) Financial support for initial DBT training. (3) Financial support for MAP Train the Trainer (4) Authorization of existing [staff] to be authorized Train the Trainers of TFCBT, CBT, Seeking Safety.”*
	**iv.** **Anticipated reduction in training resources provided by LACDMH (System Leaders).** *“Regarding training funds, [LACDMH] explained that the funds to pay for training would decrease countywide, affecting all agencies…[LACDMH] encouraged the agency to network with other provider agencies to share costs of providing training to staff.”*
**b. Difficulty utilizing PEI Funding Allocation**	**i. Underutilization of PEI funds (Agency Leaders).** *“Based on PEI claims so far in FY 2012-13, it is projected that [the agency] will only use 44% of its PEI allocation…The number of clients and the amount of claims have both decreased. The agency explained that it hopes that its new access intake program and focus on getting PEI clients from outpatient clinics will result in increased utilization. Its capacity to implement PEI services has also been challenged by staff turnover and difficulty obtaining replacement training for PEI Practices.”*
	**ii. Outreach work needs more support as it is labor intensive and non-reimbursable (Agency Leaders).** *“Lastly outreach and engagement efforts lack the appropriate reimbursement rate by DMH to cover the necessary cost to implement the services to the fullest capacity and we request COS level rate as well as request advocacy from DMH to provide PEI services with providers to serve indigent populations.”*
**c. Challenges with outcome monitoring**	**i. Data entry time and resources a challenge (Agency Leaders).** *“Staff are finding that the dashboard updating and session planning aspects of MAP are time intensive and non-billable. Managing a large number of MAP cases can make carrying regular caseloads and billing percentages significantly less in this practice. This in turn lessens the number of families we are able to serve as a whole.”*
	**ii. Outcome measures not linguistically and culturally appropriate (Agency Leaders).** *“Since a good number of our clients do not read well, we need to read measures to them. We now have Outcomes Assistants who admin the measures (after training) and most of the time they need to read the questions/statements to the clients. The Spanish version of the YOQ is not very good.”*
	**iii. Outcome measures not necessarily capturing client progress (Agency Leaders).** *“Difficulty has arisen in obtaining the PEI (end of treatment) outcomes due to the youth being sporadically released without notice. As a result our data has not shown the true progress the youth have made.”*
	**iv. Consistent completion of outcome measures is challenging (System Leaders).** *“The timing of collecting outcome measures was discussed. Agency leadership reported trying to collect measures before the last session, finding that clients often do not attend the last appointment.”*
**d. Compliance with PEI Practice claiming allowances/PEI requirements**	**i. Timeline for treatment is limiting (Agency Leaders).** * Difficulty in maintaining fidelity to the models in regard to working within established time frames of treatment because of the uniqueness of the population we serve.*
	**ii. PEI targets are different population and different treatment length than what agencies used to (Agency Leaders).** *“Having the option of only selecting from the menu of DMH-approved PEI Practices, agency staff feel as though they are stuck with “fitting round pegs into square holes.” The agency acknowledged that many of its clients have already received therapy and want to learn how to move forward.”*
	**iii. Concerns about length of treatment (System Leaders).** *“The DMH Team advised the agency to closely monitor the fidelity with which it implements PEI Practices. For example, the average length of treatment for [EBP] was higher than the countywide average. In addition, at nearly 32 sessions, the average number of sessions per client was well over what is outlined in the model of 12-16 sessions.”*
	**iv. Concerns about ancillary services billed to PEI (System Leaders).** *“A number of claiming errors were noted. The agency was also advised to cease claiming to the No EBP and Unknown EBP codes.”*
**e. Guidelines developed during implementation**	**i. Confusion about PEI Implementation Requirements (Agency Leaders).** *“Communication about how and when to do things came in waves over time, without necessarily a central way of communicating. Sometimes there were some discrepancies between two parties (differences in outcomes measure implementation between CIMH & DMH OMA).”*
**2. Perceptions of PEI Practice Implementation**	
** a. Practice Coverage**	**i. Generally, practices fit at least some of agency’s current populations appropriately.** *“Agency leadership selected PEI Practices that would fit best with its clientele. For example, it chose TF-CBT believing it would be good to address the trauma experienced by the marginalized population it serves. Although at first agency leadership was unsure if the Practice would work well because many parents have poor literacy skills, it finds the Practice a good fit for the agency.”*
	**ii. Need for more practices to fit populations not served.** *“The agency provided feedback that they need a PEI Practice that specifically addresses anxiety, and were looking into Individual CBT as an option. Unfortunately, Individual CBT is a PEI Practice that is only available to directly operated adult clinics.”*
** b. Pre-PEI supports for EBP implementation**	**i. Experience with EBPs facilitated PEI implementation.** *“With a long history and focus on treating trauma, [the agency] began implementing many Evidence Based Practices (EBPs) prior to the PEI transformation and viewed the PEI Transformation as an opportunity to grow. They are educated and involved with on-going developments of Practices through relationships with developers.”*
**c. Impact on staff**	**i. Change in therapist attitudes following initial implementation.** *“Although [agency] experienced some staff resistance to the transformation at the onset, staff has embraced the PEI program after observing improved outcomes in their clients and the agency reports an increased openness to the PEI Program among new staff.”*
	**ii. Adaptations and translations require more time and out of session work and place limits on staffing.** *“In addition, some materials for the PEI Practices are not available in the languages needed, which requires extra time and expense as staff members need to translate materials for clients.”*
**3. Types of Agency Implementation Strategies**	
**a. Practice selection**	**i. Client needs and staff capacity both considered in selection of practices.** *“The agency takes clients’ cultural and linguistic needs into consideration when determining which and how many PEI Practices to train clinicians in at each site. Although clinicians believe it would be ideal to be trained in two to three (2-3) PEI Practices to avoid being overwhelmed, most clinicians are trained in four to five (4-5) practices so that they have enough tools/skills in order for the agency to be able to provide services to all age groups and meet client needs at each site.”*
**b. Integrating clinical/funding considerations in case assignments**	**i. Prioritizing fit between practice and client needs at intake.** *“Infrastructure was created for EBP assignment in which a triage team reviews each case and assigns an EBP based on symptoms. The clinicians then assess the clients and confirm the EBP. After the practice is implemented, clinicians present each case to a multidisciplinary team and describe how treatment is tailored to the client including the client’s culture 1 month after intake, after 6 months, and after a year to the ensure appropriate treatment is provided.”*
** c. Changing staffing**	**i. Creation of new positions** *“…the agency created positions for an EBP Coordinator, Agency-Wide Administrator (AWA) for each PEI Practice, and Site Coordinators that work cohesively to ensure fidelity of the PEI Practices models.”*
	**ii. Reallocating time in existing positions.** *“Recognizing the importance of PEI Program fidelity and the clinical utility of outcome measure data, the agency designated a Clinical Supervisor to monitor the PEI Program implementation and is in the process of identifying a full time employee to oversee data collection and entry.”*
**d. Infrastructure for implementation support**	**i. Increased structured opportunities for staff and management to communicate.** *“Leadership fosters a collaborative work environment that supports two-way communication between management and clinical staff. For example, management conducted a focus group to elicit feedback from senior clinicians regarding the translation and cultural adaptations required to successfully implement the PEI Program.”*
	**ii. Use of technology to facilitate PEI implementation.** *“The agency created a user-friendly Information System to monitor and track the implementation of its PEI Program internally. Reports are provided to staff monthly that include information by clinician on clients served, EBP session counts, core units and non-core units. The system generates automated reminders and administrators follow-up with clinicians when inconsistencies or core/non-core issues arise in the reports.”*
**e. Changes to clinical supervision procedures**	**i. More supervision time devoted to monitoring compliance with PEI requirements.** *“Outcomes are also discussed in supervision. In addition, supervisors are trained in all PEI Practices and review progress notes for fidelity of implementation.”*
	**ii. Addition of practice-specific supervision.** *“With a commitment to uphold PEI Program fidelity, Clinical Managers created a discussion forum by holding PEI Practice-specific consultation groups with an emphasis on sharing successful resources and techniques among the clinical team.”*
**f. Sustainment of practices**	**i. Use of Train the Trainer model.** *“When available, [the agency] uses a Train the Trainers model and focuses on staff mastery and model fidelity rather than on increasing the quantity of PEI Practices in which a clinician is trained.”*
	**ii. Implementing strategies to deter turnover.** *“[The agency] has low staff attrition, which has been a challenge for other agencies. The agency attributes this to their supportive, strengths-based, solution-focused approach. In addition, the agency closely screens applicants for experience and training in the PEI Practices offered by the agency in order to build a strong staff that fits well with its PEI Program.”*
**g. Increase outreach and engagement efforts**	**i. New linkages in community to drive referral sources.** *“The agency makes an effort to outreach to a new client population, with two (2) staff engaged in “grass roots” outreach efforts in non-traditional locations such as libraries, churches, Head Start programs, community fairs, and local clinics. In addition, the manager of the Birth to 5 program does outreach, and a local school asked the agency to make a presentation.”*
	**ii. Using multiple strategies to increase engagement** *“The agency provides bilingual and bicultural mental health services without the use of a translator. When parents and/or caregivers seek services, they can drop their children off at the “Recreation Neighborhood Center” fully equipped with a recreation room, dance studio, and weight room…In addition, the agency demonstrates its commitment to providing grassroots services by hiring and training local community residents.”*
**h. Adapt PEI practices based on age, developmental level, and culture**	**i. Adaptations made based on age, developmental level, and culture** *“Clinicians have found it necessary to adapt the delivery of some topics by involving the client in more psycho-education activities and/or role plays. For example, after the clinician uses chalk to write words on the sidewalk depicting healthy and unhealthy relationships, the client is asked to use chalk to circle only the words describing a healthy relationship.”*

### Perceptions of system implementation strategies

Both agency and system leaders commented on the *important role of training/technical assistance* in supporting continued practice use*.* Specifically, almost all agency leaders considered the initial limited availability of therapist training an obstacle (1ai). Thus, most agencies stated that having their staff trained in multiple practices during the initial rollout was a source of pride (1aii). Regarding ongoing training, agency leaders and system leaders had different perspectives on where the burden of funding should rest. Agency leaders cited the need for continued funding from the county to maintain a trained workforce in the face of turnover (1aiii), whereas, LACDMH representatives encouraged agency leaders to plan for sustainment of PEI practices including assuming fiscal responsibility for continued training (1aiv).

Related to the lack of available trainings, agency leaders also reported that it was *difficult for them to use their full PEI funding allocation* (1bi). With only few clinicians able to attend PEI practice trainings at a time, agency leaders did not initially have the capacity to bill for these services. In addition, they did not immediately have access to client populations most likely to benefit from early intervention. As such, agencies needed to launch time-intensive outreach initiatives to cultivate appropriate referral sources, which necessitated staff time that was not eligible for reimbursement (1bii).

*Challenges with outcome monitoring* (a required change to services concurrent with the PEI practice implementation) was another major theme. Agency leaders described the burden of non-billable time for scoring and entering outcome measures (1ci) and reported that practices with greater routine progress monitoring presented more challenges, especially when delivering in-home services. They also noted that outcome measures were not available in the languages spoken by their clients and existing translations were often confusing to families particularly with limited literacy skills (1cii). Clinicians feared that outcome measures may not capture progress given language barriers and client reluctance to endorse sensitive items, such as trauma exposure (1ciii). Agency leaders further emphasized the difficulty of collecting post-treatment measures in the context of high levels of drop-out and client mobility. However, system leaders focused on increasing completion of outcome measures, and provided agencies with feedback on their rates of outcome reporting (1civ).

Regarding *compliance with PEI practice claiming allowances*, agency and system leaders both indicated concerns regarding treatment length. Clinicians expressed that they struggled to complete treatment within the recommended time frame due to clients presenting with multiple stressors, such as new trauma exposures (1di). Therapists also reported needing to slow the pace of treatment to ensure that clients understood treatment principles and could implement skills outside session. Because many agencies had previously served more severe and chronic clients, some agency leaders discussed the challenges associated with adjusting their care to fit brief models designed for early stage illness and prevention (1dii).

LACDMH representatives were concerned with *compliance with PEI requirements*. During site visits, they frequently emphasized fidelity, which was often framed in terms of compliance to PEI practice guidelines, (i.e., length of treatment and cost per client) rather clinical fidelity marked by adherence to the protocol (1diii). Time was devoted to discussion of errors in processing claims for reimbursement (e.g., claims submitted for a non-contracted PEI practice; 1div) and under- or over-utilization of PEI-allocated funds. LACDMH staff often made suggestions for remedying these issues (e.g., generating new referral streams, reviewing client eligibility).

Finally, agency leaders commented on the challenges of complying with PEI implementation requirements as these *guidelines were being developed* during implementation. Agencies reported that it was difficult to track current requirements as they were changed over time. One agency described the process as trying to “fly the PEI Transformation plane while building it.” Agency leaders expressed the need for centralized communication regarding requirements and readily-available documentation to ensure that agencies could achieve compliance.

### Perceptions of EBP implementation

Overall, agency leaders recognized that the PEI Transformation had multiple impacts on their staff and their clients. Numerous agencies reported that at least one EBP addressed some portion of their clients in terms of demographics or presenting problem (2ai). However, many also reported that practices had to be adapted (e.g., treatment pace slowed) to better fit the population; and yet some clients were still not covered by these practices (2aii). Agencies that had implemented a PEI practice prior to the transformation often had existing infrastructure and reported having since further developed their capacity to implement (2bi).

Related to *staff impact*, a concern voiced across almost all agencies was the struggle to balance clinician availability and resources with the need to serve clients. Agency leaders would have ideally offered multiple practices to maximize the range and number of clients treated, but this was not feasible due to difficulties accessing trainings and the demands of learning multiple practices. More than half of agency leaders stated that it would be optimal for clinicians to be trained in 2-3 practices so they could serve a large contingent of clients. Agency leaders also reported that therapists differed in their opinions about PEI practices. Multiple agencies reported that their clinicians were enthusiastic about using the practices, especially early career therapists with less experience. However, several leaders reported that their clinicians were concerned about brief treatment in the face of frequent client stressors, low literacy and chronically disadvantaged caregivers, which often derail continuity and necessitate longer treatment.^1^ A common narrative was that clinicians who were initially reluctant became more comfortable over time, especially as they witnessed positive client outcomes and improvements in their clinical skills (2ci). Relatedly, agency leaders reported that staff had to spend additional time in session translating and explaining concepts to non-English speaking clients, which lengthened sessions and increased workload (2cii). They expressed a need for more resources to cover this extra time and reported difficulties maintaining a culturally- and linguistically-diverse workforce.

### Types of agency implementation strategies

A major aim of the current study was to characterize the implementation strategies used by agency leaders to facilitate delivery of PEI practices. In the context of multiple EBP implementation, a theme emerged concerning *practice selection.* Although they predominantly focused on existing client needs in selecting practices, many agency leaders considered clinician preferences as well to increase the potential for sustainment (3ai). Moderately-sized and large agencies selected a greater number of PEI practices to implement than smaller agencies, likely due to their greater capacity. Once practices were selected, many agency leaders indicated that they changed their intake case assignment procedures to prioritize both clinical and fiscal considerations (3bi), assigning clients to a practice rather than basing case assignments on therapist availability.

In order to manage the transition to providing PEI practices, agency leaders reported making infrastructure changes, including *changing staffing*. Multiple agencies created new positions, such as practice coordinators or leads (3ci), or reallocating the existing staff time to new functions. For example, intake coordinators took over entering outcome data, and clinical supervisors were tasked with monitoring PEI implementation compliance (3cii). Beyond staffing considerations, some agencies instituted other types of *infrastructure for implementation support.* These included structured opportunities for line staff and management to communicate, such as focus groups, all-staff meetings, or PEI-practice consultation groups (3di). A number of agencies also utilized technology to facilitate PEI implementation, particularly through using Electronic Health Records (EHR) or information systems to monitor outcomes and remind staff to administer measures, create feedback reports, flag billing concerns, and provide example progress notes (3dii). Multiple agencies also reported addressing PEI requirements by making *changes to clinical supervision procedures*, such as devoting part of supervision to monitoring compliance (e.g., reviewing outcomes, progress notes, 3ei), or creating practice-specific supervision groups (3eii) to provide additional support on skill development.

For agencies that discussed a high investment in *sustainment of practices*, most reported adopting a train-the-trainer model (i.e., supervisors are trained to lead in-house trainings for agency employees to reduce reliance on external trainings) (3fi). Staff turnover was discussed as a perpetual problem in community mental health settings that threatened practice sustainment. Staff members trained in multiple practices are especially valuable and the transfer of even one clinician who could serve multiple client types represents a significant loss. Thus, some agencies mentioned innovative strategies to deter turnover, including seniority incentive systems, creating greater opportunities for internal advancement, and maximizing fit of therapist professional goals with the agency mission during hiring (3fii).

Many agencies reported that it was necessary to significantly *increase their outreach and engagement efforts* to generate a base of clients who would benefit from PEI services. Some agencies discussed employing innovative practices to increase referrals, such as holding mental health fairs in partnership with non-mental health agencies, offering services in non-traditional formats (e.g., summer camps, afterschool groups), and partnering with local schools and organizations (4gi). Strategies to increase client engagement were also frequently discussed, including conducting home visits and providing bilingual services. A few agencies reported offering client incentives, such as food, childcare, or transportation vouchers, or incorporating a family resource center (4gii). Once clients entered services, some agency leaders discussed the need to *adapt PEI practices based on age, developmental level, and culture*. Some practices originally developed for adults had to be adapted to be developmentally appropriate for youth (e.g., using age-appropriate words, making situations more relevant to adolescents; 4hi). Agencies that serve youth with intellectual disabilities discussed reducing emphasis on the cognitive components of practices.

#### Agency structural characteristics associated with agency implementation strategies

Use of specific implementation strategies differed by agency structural characteristics (see Table 3). Overall, moderately-sized, large, and multi-site agencies employed more implementation strategies targeting multiple levels (i.e., organization, therapist, client) in response to the system-driven implementation strategies qualitatively compared to small-sized and single-site agencies. For example, more of these agencies reported creating new positions or reallocating time in existing positions than small and single-site agencies. A substantial percentage of moderately-sized and large agencies also referenced using technology to facilitate PEI implementation, implementing practice-specific supervision, and use of train-the-trainer models as compared to the percentage of small agencies. A large percentage of small agencies reported using adaptations to treatment delivery (e.g., modifying content) as an implementation strategy whereas medium and large agencies tended to report a range of strategies. A substantial percentage of small and single-site agencies also indicated particular focus on practice selection, choosing practices that best fit the agency to preserve resources and promote sustainment.

**Table 3:** Differences in agency implementation strategies by agency structural characteristics

	Size			Centralization	
Implementation strategy	Small size (*N* = 23)	Moderate size (*N* = 42)	Large size (*N* = 17)	Single site (*N* = 47)	Multi-site (*N* = 36)
Changing staffing					
*Creating new positions*	9%	12%	29%	11%	22%
*Reallocating time in existing positions*	17%	29%	47%	21%	42%
Building infrastructure					
*Use of technology*	9%	17%	29%	17%	17%
*Structured communication opportunities*	30%	43%	41%	36%	47%
Planning for sustainment					
*Use of Train-the-trainer model*	17%	50%	71%	39%	51%
*Strategies to deter turnover*	9%	14%	12%	11%	14%
Changes to clinical supervision					
*Practice-specific supervision*	4%	29%	41%	21%	36%
*Increased time on administrative activities*	17%	21%	18%	21%	14%
Adaptations to treatment delivery	43%	29%	29%	28%	41%
Practice selection	61%	57%	47%	60%	49%
Clinical/funding considerations in service allocation	35%	24%	35%	28%	28%
Increase demand					
*New linkages for referral sources*	13%	24%	24%	17%	25%
*Strategies to increase engagement*	39%	24%	35%	32%	28%

N represents the number of agencies in the structural characteristic category. Categories are mutually exclusive. However, percentages in columns do not total to 100% because strategies were not mutually exclusive

## Discussion

Using document review of qualitative data, we identified recurrent themes in early implementation experiences within community mental health agencies undergoing a rapid, large-scale system-driven transformation providing reimbursement for the delivery of multiple practices initiated in the context of a budget crisis with the goal of avoiding shut down of agencies and substantial reduction in services. These themes related to perceptions of system implementation strategies, perceptions of practice implementation, and types of agency implementation strategies. As in previous studies, experience with EBPs was associated with improved staff attitudes and implementation quality [27]. Many of the implementation strategies utilized by agency leaders in response to the PEI transformation have been associated with success in previous implementation efforts [7, 28], particularly the creation of more structured opportunities for management and front-line staff to communicate [14, 15], generating partnerships with other service systems [29], utilizing technology [30], and the train-the trainer model [2, 8]. Overall, it appears that, when faced with a system-driven reform, agency leaders independently chose and utilized implementation strategies that are supported in the literature.

Findings also suggested that, in the absence of significant external assistance (e.g., practice developer involvement or university-community partnerships), agencies established strategies that appeared to fit within the context of their resources. Results revealed differences in organizational responses to the PEI roll-out as a function of agency structural characteristics. In general, moderate, large, and multi-site agencies were more likely to utilize a range of implementation strategies and to exhibit greater flexibility in redistributing and utilizing resources. Large organizations with a more decentralized, multi-site structure appeared better equipped to adopt multiple innovations to support implementation, perhaps because they had a greater amount of slack in their resources to prepare for changes within this major reform [16]. Single-site agencies tend to have fewer staff to designate to fully administrative roles and, thus, may need to utilize existing infrastructure (e.g., supervision) to fulfill those needs. A large percentage of smaller agencies discussed efforts to increase client engagement and adapt treatments, which may indicate that these agencies did not initially have a strong intervention-population fit and needed to utilize resources to enhance fit by modifying the practice and/or by developing a client base appropriate to the practice.

Notably, although specific PEI practices were often discussed in the site visits, they were predominately discussed in the context of PEI implementation requirements (e.g., claiming, outcome measurement). References to specific PEI practices were typically brief and varied widely across agencies, with no consensus or clear emergence of themes. This was surprising because agencies were implementing multiple practices simultaneously and practice-specific concerns have been noted in similar implementation efforts [31, 32]. It is possible that concerns regarding PEI requirements took precedence over issues related to the practices themselves given the foremost pressure to demonstrate compliance with the fiscal mandate and the urgent nature of PEI that occurred with an accelerated timeline which included three linked reforms (i.e. PEI practice delivery, expansion in patient population, and clinical outcome collection). It may be that practice-specific concerns did not emerge until after the early implementation period, once the more general concerns had been addressed.

These findings were likely shaped by both characteristics of the implementation approach and conditions of the outer context during the initial implementation of PEI. First, this *implementation was initiated by the system (LACDMH)*, in contrast to other efforts initiated by researchers, intervention purveyors, or service organizations. The system *used a reform of fiscal reimbursement policies and practices* to facilitate rapid implementation. This occurred in the context of a state budget crisis which put significant financial strain on service organizations that threatened their viability. In response, LACDMH amended and accelerated the PEI implementation plan in order to preserve the viability of agencies and availability of services to clients. As such, many of the themes that emerged related to agencies rapidly restructuring to comply with new and evolving requirements for reimbursement. Additionally, the themes reflected the context of simultaneous and rapid implementation of *multiple practices.* As such, this introduced the complexity of selecting practices to meet the characteristics of the agency workforce and target client populations. Furthermore, the themes observed were likely influenced by the characteristics of the client population served in LACDMH, which includes a predominantly ethnically- and linguistically-diverse community with relatively high levels of acuity. These client characteristics often required more intensive levels of care than intended within the prevention and early intervention focus of these services. Lastly, the EBP implementation occurred in the context of multiple, concurrent shifts in services including the target population and implementation of an outcome collection requirement.

System and agency leaders both highlighted barriers and facilitators regarding adequate training resources, ongoing funding, progress monitoring, and billing allowances, which have been identified as important themes in implementation as usual [18, 23, 33]. LACDMH staff viewed agency responses through the lens of ensuring systematic accountability to the broader goals of PEI (e.g., outcome-focused, sustainment of practices, reaching the intended population) similar to policymakers at the state and county levels in other systems [24], whereas agency leaders tended to view these factors through the lens of their local contexts and consumer needs [23, 34]. Agency leaders also emphasized the fit of individual practices with organizational, therapist, and client characteristics. In sum, system and agency leaders focused on the most immediate pressures facing each level of stakeholder. LACDMH system leaders’ focus on contractual compliance reflected their responsibility to ensure accountability in spending public funds. Agency leaders had to attend to the immediate viability of their programs and ensuring continuity of care for their vulnerable clients.

Given that the EBP implementation described in the present study occurred within a unique and rapidly-changing environment, it is difficult to know the extent to which the findings are attributable to the agencies themselves or the external context. It is also a challenge to estimate the relative importance or impact of each implementation strategy on the outcomes. As this is a neutral, observational study in which there was no manipulation of the variables, this is a limitation of the data and should be considered when interpreting findings. As dissemination and implementation science continues to progress, it would be beneficial to conduct dismantling studies of implementation strategies as well as prospective studies of implementation to determine unique and defined benefits of different implementation strategies. It is imperative to customize implementation strategies to an organization’s needs rather than using a prescriptive, one-size-fits-all approach, and more work is needed to determine how to tailor individual strategies [35].

There are important limitations to note regarding this study. Utilization management reports furnished data regarding factors at the organizational level (e.g., unique client count). These measures were used as proxies that were intended to provide information regarding organizational characteristics and therefore do not directly measure features of agencies, such as their staffing resources. The TASVs were also prepared by a third-party organization and, as such, are not a direct representation of agency and LACDMH perspectives. Given the length of time that has passed between these meetings and the current study as well as high turnover rate of agency staff since the meetings occurred, we were not able to independently validate these documents. These documents were also intended to provide a record of the major points discussed during the visit and, therefore, may not provide the level of detail and consistency regarding themes that would be provided by primary data collection. Surveys and interviews directly querying therapists and program managers about organizational factors and their perceptions of PEI implementation are currently being administered and can provide this level of analysis. Lastly, given that this is retrospective, cross-sectional study that occurred within a unique context, it is important to consider that the conclusions may not be generalizable to other contexts undergoing implementation efforts, particularly given that implementation was marked by a time-sensitive budget process.

## Conclusions

Despite these limitations, there are a number of important implications of this study, which add to the limited body of research regarding system-driven implementation efforts of multiple EBPs. Overall, this study highlights the complexity involved in rapid large-scale system reform and suggests that there are unique factors to consider at each level in the inner and outer context that may facilitate successful implementation. At the agency level, it is important to select and utilize implementation strategies that fit well with the organization, such as making changes to triage procedures or re-organizing staff positions. At the systems-level, it is important to facilitate collaboration between agencies and disseminate strategies that agencies find beneficial. It may also be important for leaders at both the agency and the systems levels to consider agency characteristics when selecting implementation strategies as these may affect the feasibility and fit of strategies, such as utilizing technology or hiring new staff. Whereas larger agencies may have the capacity to enact these implementation strategies independently, smaller agencies might require additional supports. Finally, simultaneous implementation of multiple practices requires shifts in care delivery and requirements associated with individual EBPs (e.g., outcome measures, claiming compliance) may pose challenges for agencies to consider beyond the clinical components of delivering the intervention itself. This is particularly salient as system leaders may rank implementation priorities differently than agency leaders. In preparing agencies to deliver multiple EBPs, it is important to choose practices that fit the agency’s structure and organization and the client population, but it may be equally or even more important to consider streamlining implementation requirements and providing initial and ongoing support in meeting requirements across agencies.

## Abbreviations

CBITS: Cognitive Behavioral Intervention for Trauma in Schools
CPP: Child-Parent Psychotherapy
EBP: Evidence-based practice
EHR: Electronic Health Record
EPIS: Exploration, Preparation, Implementation, Sustainment framework
LACDMH: Los Angeles County Department of Mental Health
MAP: Managing and Adapting Practice
MHSA: Mental Health Services Act
PEI: Prevention and Early Intervention
PSVQ: PEI provider pre-site visit questionnaire
SS: Seeking Safety
TASV: PEI technical assistance site visit summary report
TAY: Transition-age youth
TF-CBT: Trauma-Focused Cognitive Behavioral Therapy
Triple P: Triple P Positive Parenting Program

## Footnotes

1 Session limits were put into place for each practice by LACDMH based on developer feedback.
